# Comparison of stress and coping mechanisms among family members of patients abusing licit and illicit substances

**DOI:** 10.1192/j.eurpsy.2023.614

**Published:** 2023-07-19

**Authors:** S. Mehta, A. Jaiswal, S. Khattri, S. Garg

**Affiliations:** Psychiatry, Sri Guru Ram Rai Institute of Medical & Health Sciences, Dehradun, Uttarakhand, India

## Abstract

**Introduction:**

Addiction has long been recognized as a disease that may have a dramatic influence on the addicted individual’s family members, manifesting as stress or trauma-related physical and psychological symptoms, resulting in increase in the usual family’s use of health-care services. There is little research available to identify and explore problems of families associated with such patients. In this study, we will evaluate stress among family members of substance abuse patients and try to focus on how these family members are dealing with these stress factors. The study will also compare different types of coping mechanisms among family members of patients taking licit and illicit substances.

**Objectives:**

To identify stress among families of patients abusing licit and illicit drugs and assess and compare their coping mechanisms.

**Methods:**

175 family members of patients with licit substance abuse cases and 175 family members of patients with illicit substance abuse were taken for study after informed consent. Stress among participants was evaluated using Symptom Rating Test and The Depression Anxiety and Stress Scales (DASS). Coping Questionnaire (CQ) and Breif COPE was used to assess coping among them.

**Results:**

The study demonstrated that the total number and severity of symptoms, including psychological and physical symptoms, are found lesser in caregivers (participants) of patients abusing licit substances than in patients abusing illicit drugs. These symptoms among family members grow as the patient’s age rises. Symptoms in participants are more if their patient is female as compared to male. And also, total symptoms are more if they are living in nuclear family (Table 1). In our results, total coping and engaged coping mechanisms in family members are found to be not significant as per drug type and remains the same for both licit and illicit drugs abusing patients’ families. Avoidant coping mechanisms in family members are more if their patient is female and in nuclear family.

**Table 1:** 

**symp1**	**Coefficients**	**Std. Err.**	**P Value**
age	0.005*	0.001	0.000
drug_type	-0.114*	0.027	0.000
female_d	0.230*	0.028	0.000
nuclear_d	0.124*	0.027	0.000

**Conclusions:**

In our study, it was clearly noticed that the total symptoms, both physical and psychological, were found more in family members of patients abusing illicit drugs. Impact of substance abuse related problems is found more in female members compared to male members of family.

**Disclosure of Interest:**

None Declared

